# The Greater Impact of Paternal, Compared to Maternal, Hereditary Background on Depressive-Like Behavior in Wistar Kyoto Rats with Different Amino Acid Metabolism in the Pup Brain

**DOI:** 10.3390/ijms24044199

**Published:** 2023-02-20

**Authors:** Tsubasa Ihara, Mizuki Hamada, Mitsuhiro Furuse

**Affiliations:** Laboratory of Regulation in Metabolism and Behavior, Faculty of Agriculture, Kyushu University, Fukuoka 819-0395, Japan

**Keywords:** major depression, heredity, Wistar Kyoto rats, forced swimming test, open field test, amino acids

## Abstract

In the pathogenesis of depression, heredity is believed to be a major factor. However, the mechanism by which heredity contributes to the onset of depression is not fully understood. Wistar Kyoto (WKY) rats have been used as an animal model for depression because of their increased depression-like behavior compared to Wistar (WIS) rats. In the present study, pups crossbred from WKY × WIS rats were used to evaluate locomotor activity in an open field test (OFT) and depression-like behavior in a forced swimming test (FST), with a focus on amino acid metabolism. Pups in the WKY♂ × WKY♀ group showed lower locomotor activity in the OFT and higher depression-like behavior in the FST than those in the WIS♂ × WIS♀ group. In addition, multiple regression analysis showed that the paternal strain had a greater effect than the maternal strain on locomotor activity and depression-like behavior in OFT and FST, respectively. Several amino acids in the brainstem, hippocampus, and striatum were significantly decreased through the influence of the WKY paternal strain, but not the WKY maternal strain. Based on these data from comparing WKY and WIS rats, we hypothesize that the hereditary effects of the WKY paternal strain on behavioral tests are partially caused by dysregulation of the amino acid metabolism in the brain.

## 1. Introduction

Depression is a major problem in modern society; it was reported in 2021 that approximately 280 million people were suffering from depression [1]. Therefore, early clarification of the pathogenesis of depression is required to improve the quality of life throughout the world.

In the pathogenesis of depression, heredity is believed to be a major factor [2]. However, the mechanism by which heredity contributes to the onset of depression is not fully understood. It is well known that women suffer from major depression more often than men, and an estimated 10–25% of women and 5–12% of men experience major depression in their lifetime [3,4]. In addition, previous studies have shown that reactions to antidepressant treatments differ between men and women [5,6]. These data suggest that there are sex differences in the pathology of depression. There was a significant association between antenatal depression and adverse birth outcomes and low birth weight, and preterm birth was also confirmed as a significant effect of perinatal depression on adverse infant health outcomes, such as malnutrition [7].

Despite this, in most previous studies, male animals have been used exclusively as animal models of depression-like behavior. However, for the above reasons, female animals should also be used to examine the cause of sex differences in depression.

Wistar Kyoto (WKY) rats are a strain bred from Wistar (WIS) rats and are used as a control animal for spontaneously hypertensive rats. However, previous studies on stress ulcers have shown that WKY rats are hyper responsive to stress [8,9,10]. In addition, WKY rats display a high level of immobility in the forced swimming test (FST) compared to other inbred strains [11,12]. Therefore, WKY rats are considered an appropriate animal model for depression. Several studies have demonstrated that monoamine and free amino acid levels in a variety of brain regions differ between WIS and WKY rats [13,14], suggesting that depression-like behavior in WKY rats may be associated with dysfunction in monoamine or amino acid metabolism in the brain. In the case of dysfunction in amino acid metabolism, dietary treatments of whole egg protein [15] or amino acid [16] to WKY rats exerts an antidepressant-like effect.

In the present study, WKY rats were used as a model of depression-like behavior and WIS rats were used as a control. Pups from WKY × WIS crossbreeding were used to evaluate locomotor activity in the open field test (OFT) and depression-like behavior in the FST. To explain the behavioral changes, amino acid metabolism was evaluated, as amino acids play important roles in the brain and body [17]. Using this approach, the effects of paternal vs. maternal hereditary background on locomotor activity or depression-like behavior in pups, along with differences based on sex, were also investigated. Previously, we determined the concentrations of free amino acids in the prefrontal cortex, striatum, thalamus, hypothalamus, hippocampus, brainstem, and cerebellum of WIS rats and WKY rats [18]. It was clear that changes in amino acids were different dependent upon brain regions, and nine amino acids in the hippocampus, seven amino acids in the striatum, and three amino acids in the brainstem were lower in WKY rats compared with WIS rats. Accordingly, we examined whether these three sites were likely to be influenced by the parents. 

## 2. Results

### 2.1. Maternal Behavior

No significant difference in total maternal behavior was observed between WIS rats (360 ± 21) and WKY rats (323 ± 15).

### 2.2. Body Weight

**Figure 1:**
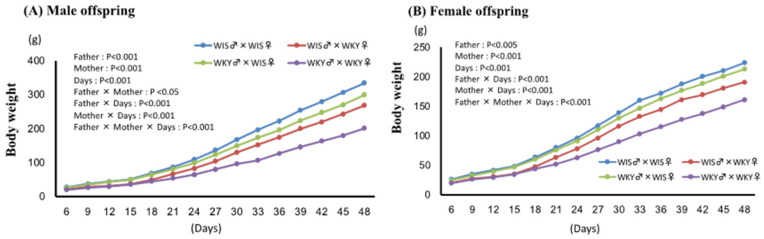
Effect of paternal or maternal strain on body weight of offspring. Body weight in the male (**A**) and female (**B**) pups was examined every 3 days and expressed as means ± SEM. Days refer to postnatal day after delivery/birth; WIS♂ × WKY♀ refers to pups of male Wistar (WIS) and female Wistar Kyoto (WKY) rats (*n* = 6).

Changes in body weight of pups are shown in Figure 1A,B. Body weight significantly increased with time in both sexes, but pups with paternal or maternal WKY rat showed significantly lower body weight than WIS♂ × WIS♀ pups. In both sexes, significant interactions were observed between paternal and maternal strains, suggesting that the effects of parental strain were synergistic. Furthermore, significant interactions were observed between paternal and maternal strain and time, suggesting that the differences in body weight between groups increased as time passed. In addition, significant interactions were observed among paternal strain, maternal strain, and time, suggesting that the effects of paternal strain emerged later than the maternal.

### 2.3. OFT

**Figure 2:**
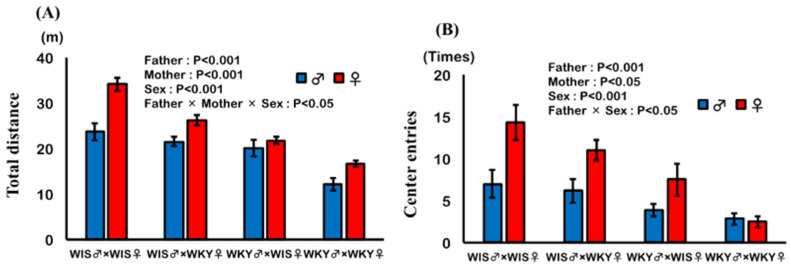
Effect of paternal or maternal strain and sex of pups on results of open field test. Total distance (**A**) and the number of entries to the central area (**B**) were analyzed and expressed as mean ± SEM. Pups were 56–57 days old; WIS♂ × WKY♀ refers to pups of male Wistar (WIS) and female Wistar Kyoto (WKY) rats (*n* = 5–6).

The effects of the paternal or maternal strain and sex of pup on the total distance and the number of center entries are shown in Figure 2A,B. Paternal and maternal strains had significant effects, and pups with WKY rat as a parent showed significantly decreased total distance and number of center entries. Furthermore, sex of pup exhibited a significant effect; male pups showed significantly decreased total distance and number of center entries. In total distance, a significant interaction was observed among paternal strain, maternal strain, and sex, suggesting that the effect of parental strain was additive in female pups and synergistic in male pups. In a number of center entries, a significant interaction was observed between paternal strain and sex, suggesting that the effect of paternal strain was greater in female pups. In addition, the multiple regression analysis showed that the coefficient of paternal strain was greater than that of the maternal strain in both total distance and center entries, judging from the constants for paternal and maternal strains (distance (m) = 35.820 (SE = 2.723) − 8.942 (SE = 1.039) × paternal strain − 5.992 (SE = 1.039) × maternal strain + 5.668 (SE = 1.039) × Sex (R^2^ = 0.754, RMS = 3.560); number of center entries (times) = 13.085 (SE = 2.783) − 5.460 (SE = 1.062) × paternal strain − 2.544 (SE = 1.062) × maternal strain + 3.877 (SE = 1.062) × sex (R^2^ = 0.505, RMS = 3.638)).

### 2.4. FST

**Figure 3:**
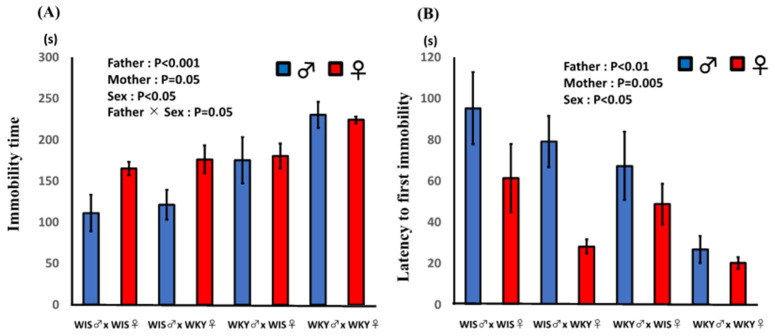
Effect of paternal or maternal strain and sex of pups on the results of the forced swimming test. (**A**) Immobility time and (**B**) latency to first immobility. Total immobility time and latency to first immobility were analyzed and are expressed as mean ± SEM. Pups were 60–61 days old; WIS♂ × WKY♀ refers to pups of male Wistar (WIS) and female Wistar Kyoto (WKY) rats (*n* = 6).

The effects of paternal or maternal strain and sex of pup on (A) the total duration of immobility and (B) latency to first immobility are shown in Figure 3. Paternal and maternal strain both had significant effects, and pups with a WKY rat as a parent showed significantly longer immobility. Furthermore, the sex of pup exhibited a significant effect; male pups showed a significantly shorter duration of immobility. A significant interaction between paternal strain and sex of pup was observed, suggesting that the effect of paternal strain was stronger in male pups. Paternal and maternal strain both had significant effects, and WKY strain showed significantly shorter latency to first immobility. Female pups exhibited a shorter latency to first immobility compared with male pups. In addition, the multiple regression analysis showed that the coefficient of paternal strain was greater than that of maternal strain. Immobility time (S) = −1.070 (SE = 33.964) + 58.712 (SE = 12.837) × paternal strain + 30.062 (SE = 12.837) × maternal strain + 27.166 (SE = 12.837) × sex (R^2^ = 0.412, RMS = 44.470). On the other hand, the coefficients for latency to first immobility were not greatly different between fathers and mothers. Latency to first immobility (S) = 176.630 (SE = 22.654) −25.163 (SE = 8.562) × paternal strain −29.552 (SE = 8.562) × maternal strain −27.472 (SE = 8.562) × sex (R^2^ = 0.412, RMS = 29.661).

### 2.5. Amino Acids

The concentrations of free amino acids in the brainstem, hippocampus, and striatum of pups are shown in Table 1, Table 2 and Table 3, respectively.

In the brainstem, the concentrations of L-aspartic acid (Asp), L-serine (Ser), γ-aminobutyric acid (GABA), and L-valine (Val) were significantly influenced by maternal strain, and all of them decreased in pups with WKY rats as a mother. Significant interactions were found among paternal strain, maternal strain, and pup sex for L-Asp, L-Ser, and GABA, suggesting that amino acid levels in pups were modified by the combination of parent strain and paternal strain. The concentration of L-histidine (His), L-arginine (Arg), taurine (Tau), L-alanine (Ala), and L-isoleucine (Ile) were significantly influenced by paternal and maternal strain. While L-His and Tau increased in pups that had WKY rats as a parent, L-Arg, L-Ala, and L-Ile decreased. Significant interactions existed among paternal strain, maternal strain, and pup sex in L-Arg and Tau, and between paternal strain and pup sex in L-His and L-Arg, suggesting that these amino acid levels in pups were modified by the combination of parent strain and paternal strain. L-Tyrosine (Tyr) and L-methionine (Met) were significantly influenced by maternal strain and sex of pup. L-Tyr and L-Met decreased in pups with WKY rats as a mother. While L-Tyr decreased in females, L-Met decreased in males. A significant interaction was observed for L-Tyr between maternal strain and sex of pup, suggesting that the effects of maternal strain were greater in female pups. A significant interaction was found for L-Met between paternal and maternal strain, suggesting that L-Met decreased when either parent was WKY. The concentration of L-phenylalanine (Phe) was significantly influenced by paternal strain, and L-Phe decreased in pups with WKY rats as a father. Paternal and maternal strain and sex of pup significantly influenced the concentration of L-Ile. L-Leucine (Leu) decreased in female pups and in those with WKY rats as a parent. A significant interaction was observed between paternal strain and sex of pup in L-Leu, suggesting that the effect of paternal strain was greater in male pups. Significant interactions among paternal strain, maternal strain, and pup sex were observed for L-glutamine (Gln), suggesting that this amino acid level in pups is modified by the combination of parent strain and paternal strain. No significant effects for D-Ser were detected among any treatment.

In the hippocampus, the concentration of L-Asp, GABA, and L-Met were significantly influenced by paternal strain and sex of pup and all of them decreased in male pups and in those with WKY rats as a father. The concentration of D-Ser, L-Ser, and L-Ala were significantly influenced by paternal and maternal strain and sex of pup, and all of them decreased in male pups and in those with WKY rats as the mother. The concentration of L-Gln, L-Arg, L-Phe, L-Ile, and L-Leu were significantly influenced by sex of pup, and all of them decreased in male pups. Significant interactions for L-Gln and L-Arg were found among paternal strain, maternal strain, and sex of pup, suggesting that L-Gln and L-Arg in male pups decreased when either parent was WKY. The concentration of L-Val was significantly influenced by paternal strain, and L-Val decreased in pups with WKY rats as a father. A significant interaction among paternal strain, maternal strain, and sex of pup indicates that L-Val decreased when the father was WKY and the mother was WIS. For L-His, significant interactions between paternal strain and maternal strain and among paternal strain, maternal strain, and sex of pup were detected. L-His in male pups was decreased when either parent was WKY but increased when both parents were WKY. A significant interaction for Tau was found among paternal strain, maternal strain, and sex of pup, suggesting that Tau in male pups decreased when either parent was WKY. No significant effects for L-Tyr were detected among any treatment.

In the striatum, the concentration of L-Asp, L-Ser, GABA, L-Tyr, L-Val, and L-Met were significantly influenced by paternal strain, and all of them decreased in pups with WKY rats as a father. Significant interactions for L-Ser, L-Tyr, and L-Val between paternal strain and sex of pup indicated that these amino acids were decreased in female pups when the father was WKY. The concentration of D-Ser was significantly influenced by paternal and maternal strain and sex of pup, and D-Ser decreased in male pups and in those with WKY rats as either parent. Significant interaction was observed between paternal strain and sex of pup in D-Ser, suggesting that the effect of paternal strain was greater in female pups. The concentration of L-Gln, L-His, L-Arg, and Tau were significantly influenced by paternal strain and sex of pup. All of them decreased in pups with a WKY rat as a father. While L-Gln, L-His, and Tau decreased in male pups, L-Arg decreased in female pups. A significant interaction between paternal strain and sex of pup was observed in L-Gln, L-His, and Tau. Significant interactions for L-Gln, L-His, and Tau between paternal strain and sex of pup suggested that these amino acids were decreased in female pups when the father was WKY.

## 3. Discussion

In the present study, pups from the WKY♂ × WKY♀ group showed lower total distance and center entries in OFT than those of the WIS♂ × WIS♀ group. This result was consistent with previous studies in which WKY rats showed low locomotor activity [14,19]. Three-way ANOVA showed a significant effect for paternal and maternal strains and sex of pup on total distance and center entries. In center entries, especially, a significant interaction was observed between the paternal strain and the sex of the pup, suggesting that female pups were greatly influenced by the paternal strain. In addition, multiple regression analysis showed a greater effect of paternal strain judging from the coefficient between paternal and maternal strains. According to these results, paternal strain has a greater hereditary effect than maternal strain on locomotor activity, and the effect is greater in female pups. In FST, pups from the WKY♂ × WKY♀ group showed higher immobility and greater depression-like behavior. This result was similar to those obtained in previous studies [14,18]. Three-way ANOVA showed a significant effect for paternal and maternal strain and sex of pups on immobility time. A significant interaction was observed between paternal strain and sex of pup, suggesting that male pups were greatly influenced by paternal strain. This result differed from the OFT, in which female pups were influenced by paternal strain. In addition, multiple regression analysis showed a greater effect of paternal strain. According to these results, paternal strain has a greater hereditary effect on depression-like behavior, and the effect is greater in male pups. Judging from both behavioral tests, paternal strain greatly influenced pup behavior, but sex differences existed between the two tests applied here. On the other hand, OFT was extended to assess a wider range of behavioral features and psychiatric conditions such as anxiety-like behavior. According to Rosso et al. [20], it is difficult to conclude that the results obtained by OFT fitted to the parameter for anxiety-like behavior. They revealed that among the most popular tests for anxiety assessment, only two test measures—time in open arms in elevated plus maze and time in light compartment in light–dark box paradigms—appear to be sensitive in the representation of anxiogenic/anxiolytic traits. The results obtained with OFT will need further detailed examination in future.

While no significant strain difference was observed in maternal behavior, results did reveal that maternal strain has a significant influence on body weight gain of pups. Milk amino acids, both free and as constituents of protein, play an important role in the growth and development of offspring. In particular, free amino acids are important because they act as immediate nutrients. We confirmed that nutrition in early infancy may affect later health and behavior of offspring [21,22]. The differing growths among treatments may be explained by milk constituents, since WKY rats have differences from WIS rats in the amounts of free amino acids in milk [23]. In contrast to body weight, paternal strain has a greater effect than maternal strain on locomotor activity and depression-like behavior, as seen in OFT and FST.

In many previous studies, only male rats were used as a model of depression-like behavior [11,13,14]. However, considering the fact that the incidence of depression in females (15%) is almost twice as high as in males (8%) [3,4], female rats should be used to examine the causes of sex differences in depression. In this study, the hereditary effect of paternal strain in FST was more remarkable in male pups, but in OFT it was more remarkable in female pups. Judging from these data, WKY could represent an important animal model to study sex differences in the pathology of depression. 

Free amino acid analysis revealed that many amino acids were significantly influenced by parental strain and sex of pup. In this experiment, we investigated the concentration of free amino acids in three regions of the brain. As a result, it was revealed that the maternal influence was slightly stronger in the brainstem, and that paternal influence strongly appeared in the hippocampus and striatum.

Previous antidepressants have focused on monoamine metabolism in the brain. However, free amino acids and their metabolites can be used as neurotransmitters, i.e., L-glutamic acid (Glu), L-Asp, GABA, and glycine (Gly), and as precursors of monoamines, i.e., L-Trp and L-Tyr. Furthermore, some amino acids, i.e., L-Asp [24], L-Arg [25], L-Ser [26], and D-Ser [27], have sedative or hypnosis-like effects under stressful conditions in neonatal chicks or mice. In this study, the concentration of amino acids, such as L-Asp, GABA, and L-Ser, in the brainstem, hippocampus, and striatum were decreased in pups that had a WKY rat as a parent. These results are consistent with previous studies [14,18]. Interestingly, different paternal and maternal involvement in different brain regions suggests that alterations occurred in the metabolic pathways of these amino acids. The WKY rats may be abnormal in metabolism and neurotransmission with these amino acids. L-Ser was affected by the WKY parent at all three sites, but D-ser was unchanged only at the brainstem. D-Ser is synthesized from L-ser by a serine racemase, but the D-ser/L-ser ratio in the brainstem was lower than in the other two sites. The activity of serine racemase in the brain stem was originally low, and it is possible that the influence of WKY parents was difficult to distinguish. For other amino acids, the manifestation and response of genetic effects differed in different brain regions. It is difficult to explain amino acid metabolism in a unified manner, and it is possible that amino acid metabolism changes specifically in each part of the brain depending on the influence of the sex of the parent and offspring. 

In addition, the concentration of amino acids, such as L-Asp in the hippocampus and striatum, L-Ser in the striatum, and GABA in the hippocampus and striatum, were significantly influenced only by paternal strain. The effect of paternal strain on behavioral tests may be the result of changes in the metabolism of these amino acids. New antidepressants which are effective against drug-resistant depression may be developed through further analysis of these amino acids.

There were some limitations to note with the present study. Firstly, only the concentration of amino acids was evaluated in this study, and not their release into the synaptic cleft. Therefore, future studies should evaluate alterations in the release of these neurotransmitters into synaptic clefts that are caused by hereditary effects. Secondly, female rats were used in this study. Previous studies report that the depression-like behavior in female rats is subject to their estrus cycle [28], which could apply to the female rats used in this study. However, the SEM for almost all behavioral tests and amino acid analyses were smaller in females than in males, suggesting that female rats are an effective animal model for depression in the experimental conditions used here. Finally, WKY is an established inbred rat strain, and genes on the autosome are identical whether inherited from the father or mother. However, monoallelic expression of imprinted genes must be considered. Imprinted genes are inherited in duplicates, similar to other genes; however, they are epigenetically marked and solely (or predominantly) expressed from one of the parental inherited alleles [29], and, in fact, some genes are preferentially paternally inherited [30]. Some imprinted genes highly expressed in the brain are postulated to affect neurodevelopment and ongoing brain function [31]. In addition, sex chromosome genes may correlate with the heredity of depression. For example, various studies have demonstrated that SRY (the sex-determining region on the Y chromosome) regulates the dopamine pathway in vitro and in vivo in males [32,33,34]. The pathogenesis of depression may be elucidated through further studies focusing on these imprinted and sex chromosome genes.

## 4. Materials and Methods

### 4.1. Animals 

Seven-week-old WIS (6 female and 6 male) and WKY rats (6 female and 6 male) (Charles River Japan, Yokohama, Japan) were examined. Rats were housed 3 per cage with free access to a standard diet for laboratory rodents (MF, Oriental Yeast, Tokyo, Japan) and water. A 12 h light/dark cycle (lights on at 08:00, lights off at 20:00), a room temperature of 23 °C, and humidity of 60% were maintained throughout the study. The study was performed according to the guidelines for Animal Experiments in the Faculty of Agriculture and in the Graduate Course of Kyushu University (A28-184-1), and in accordance with Law No. 105 and Notification No. 6 of the government of Japan.

### 4.2. Experimental Procedure 

To examine the effect of paternal vs. maternal hereditary background on locomotor activity or depression-like behavior of pups and its differences based on sex, the following experiment was planned: WKY♀ × WIS♂, WKY♀ × WKY♂, WKY♂ × WIS♀, and WIS♂ × WIS♀, three pairs each, were mated to generate pups in Plexiglas transparent cages (44 × 20 × 21 cm, one pair/cage). One week after the start of mating, female rats were separated from male rats. The day of birth was considered PPD0; on the morning of PPD0, litters were culled to 6 (3 males and 3 females) pups and the mother. 

Maternal behavior was recorded from PPD1 to PPD6. After maternal behavior observation, the body weights of pups were recorded every 3 days (PPD6 − PPD48). All pups were weaned at PPD21 and separated into groups with 3 rats/cage. After weaning, the pups had free access to food that the mother was given, as well as water.

**Figure 4:**
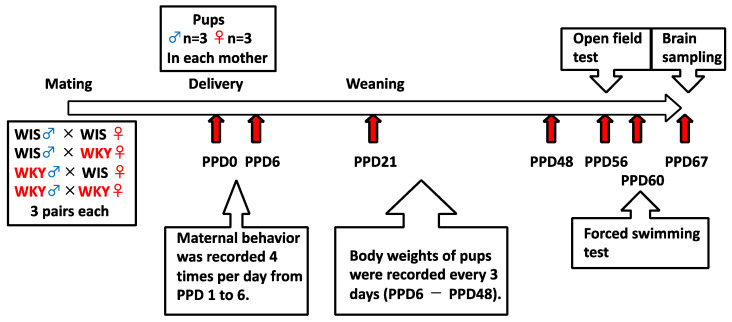
Experimental design of this study.

The OFT was performed on PPD56 and PPD57, and the FST was performed on PPD60 and PPD61. Behavioral tests were conducted during light periods, and behavior during the tests was recorded by video capture system. One week after FST, all rats were euthanized under anesthesia with isoflurane (Escains, Mylan, Osaka, Japan). The brains were quickly removed from the skulls, and the brainstem, striatum, and hippocampus were dissected. Samples were frozen in liquid nitrogen and stored at −80 °C until analysis. The experimental design of the present study is demonstrated in Figure 4.

### 4.3. Behavioral Tests 

The OFT and FST were performed by randomly selecting 2 rats each from 3 males and 3 females raised by each mother rat. As a result, there were 6 male pups and 6 female pups in each group of WKY♀ × WIS♂, WKY♀ × WKY♂, WKY♂ × WIS♀, and WIS♂ × WIS♀.

#### 4.3.1. OFT

Here, OFT was performed to evaluate locomotor activity in a novel environment using a square arena (length 90 cm, width 90 cm, and height 45 cm), which was made of black-colored wood. The test was performed under light conditions of 100 lx for 5 min. At the beginning of each test, the rat was transferred to the center of the open field area from its home cage. After each trial, the arena was cleaned with 10% ethanol solution to standardize the conditions for all tests. Open field behavior was analyzed using ANY-maze software (Stoelting Co, Wood Dale, IL, USA) by dividing the field into 25 squares (18 × 18 cm). The total distance moved and the number of entries into the central area (3 × 3 square areas) were measured.

#### 4.3.2. FST

The FST was performed to evaluate depression-like behavior in the rats according to the method described in a previous report [35] with some modifications. The total duration of immobility was used as an index for depression-like behavior. This test consisted of two exposure sessions, i.e., a pre-test session and a main-test session. In the pre-test session, each rat was individually placed in an acrylic cylinder (30 cm in diameter, 45 cm high) with water maintained at 25 °C at a depth of 30 cm for 15 min. After 24 h, the main-test session was performed in which each rat was returned to the acrylic cylinder containing water in the same manner as the pre-test session, and the behavior in water was recorded for 5 min by video capture system for each trial. After each session, the water was wiped away with dry paper and the rats were returned to their home cages. For each trial, the water in the acrylic cylinder was replaced with fresh to standardize the test conditions. The total duration of immobility was measured manually by a rater blinded to the experimental group. Rats were considered immobile when they floated motionless or made only small movements to keep their head above water.

#### 4.3.3. Maternal Behavior

Maternal behavior was recorded 4 times per day, including 3 periods during the light cycle (11:00, 14:00, 17:00 h) and once at the beginning of the dark cycle (20:00 h), from PPD 1 to 6. Within each observation session, the behavior of each female at that specific moment was scored every 3 min (25 observations in 4 periods per day = 100 total observations/mother/day). The following behaviors were recorded: high crouch posture (mother nursing pups in an arched-back posture); low crouch posture (mother nursing pups in a “blanket” low arched back posture); supine posture (a passive posture in which the mother is lying on her back or side while the pups nurse); licking the pups (licking the surface of their bodies and their anogenital regions); nest building (piling sawdust were the pups laid); retrieving (retrieving pups to the nest); maternal off the nest (the lactating female is out of the nest); and self-grooming (mother grooms herself). The maternal care score was calculated by summing the behaviors directed toward the pups (licking + breastfeeding postures + nest building + retrieving) from all recording periods. The data are reported as the number of observations recorded for each behavior (maximum of 600 times) [36,37,38] and were averaged among the females within the groups. 

### 4.4. Free Amino Acid Analysis

Amino acid content in different brain regions was analyzed according to a previously described method [39]. The content of both L- and D-amino acids was measured using ultraperformance liquid chromatography (UPLC) (the Acquity™ UPLC system comprised a Waters Binary Solvent Manager, a Waters Sample Manager, and a Waters FLR Detector) with an ACCQ-TAG™ ULTRA C18 1.7 μm, 2.1 × 100 mm column (Waters Corporation, Milford, MA, USA). The excitation and emission wavelengths for the fluorescent detection of amino acids were 350 nm and 450 nm, respectively. The system was operated at a flow rate of 0.25 mL/min at 30 °C. The UPLC gradient system (A = 50 mmol/L sodium acetate (pH 5.9), B = methanol) was 10–20% B over 3.2 min, 20% B for 1 min, 20–40% B over 3.6 min, 40% B for 1.2 min, 40–60% B over 3.8 min, 60% B for 1 min, and 60–100% B over 0.01 min. Just before the UPLC analysis, each sample (10 μL) was transferred to a UPLC tube and N-acetyl-L-cysteine/ophthaldialdehyde (20 μL) and borate buffer (70 μL) were added; then, the sample was left for 2 min in a dark room. The same method was used for standard solutions containing L- and D-amino acids (Asp, Glu, Ser, Gln, His, Arg, Ala, Tyr, Val, Met, Phe, Ile, and Leu), Gly, Tau, and GABA. The amino acid concentrations in the brain were expressed as nmol/mg of wet tissue. 

### 4.5. Statistics 

All data are expressed as mean with SEM. Body weight was analyzed by a repeated-measures three-way ANOVA. The number of center zone entries and total distance in the OFT, the total duration of immobility in the FST, and free amino acid concentrations were analyzed using three-way ANOVA; significance was set at *p* < 0.05. Multiple regression analysis was applied to the OFT and FST for the variables of parental strain and sex of pups (strain: WIS rat =1, WKY rat = 2, sex: male =1, female = 2). All analyses were performed with StatView (version 5, SAS Institute Cary, USA, SAS, 1998). Outlying data from the behavioral tests and amino acid analysis were eliminated using Thompson’s test criterion for outlying observations (*p* < 0.01). Although several combinations of interactions were determined, only interactions indicating significant effects are provided in the relevant tables and figures.

## 5. Conclusions

Taking together the data mentioned above, the hereditary effects of the paternal strain on behavioral tests are stronger than those of maternal strain. Through paternal studies focusing on the dysregulation of the amino acid metabolism in the brain, more effective treatments for major depression may be developed.

## Figures and Tables

**Table 1:** Effect of paternal or maternal strain and sex of offspring on free amino acid concentration in the brainstem.

Strain	WIS♂ × WIS♀		WIS♂ × WKY♀		WKY♂ × WIS♀		WKY♂ × WKY♀		*p* Value			
Sex	Male	Female	Male	Female	Male	Female	Male	Female	Father	Mother	Sex	Interaction
L-Asp	2.83 ± 0.08	2.58 ± 0.12	2.42 ± 0.07	2.50 ± 0.06	2.44 ± 0.12	2.66 ± 0.11	2.53 ± 0.04	2.31 ± 0.05	NS	*p* < 0.01	NS	*p* < 0.01 (Father × Mother × Sex)
D-Ser	0.046 ± 0.004	0.048 ± 0.007	0.050 ± 0.003	0.052 ± 0.002	0.048 ± 0.001	0.049 ± 0.002	0.046 ± 0.004	0.053 ± 0.002	NS	NS	NS	NS
L-Ser	0.554 ± 0.02	0.497± 0.02	0.476 ± 0.01	0.472 ± 0.006	0.491 ± 0.024	0.509 ± 0.02	0.481 ± 0.006	0.448 ± 0.005	NS	*p* < 0.01	NS	*p* < 0.05 (Father × Mother × Sex)
L-Gln	4.33 ± 0.17	4.04 ± 0.11	3.89 ± 0.12	4.03 ± 0.06	4.06 ± 0.13	4.05 ± 0.07	4.25 ± 0.03	3.99 ± 0.08	NS	NS	NS	*p* < 0.05 (Father × Mother × Sex)
L-His	0.749 ± 0.08	0.940 ± 0.02	0.943 ± 0.07	0.950 ± 0.04	1.00 ± 0.06	0.932 ± 0.07	1.14 ± 0.01	1.07 ± 0.03	*p* < 0.01	*p* < 0.01	NS	*p* < 0.05 (Father × Sex)
L-Arg	0.502 ± 0.04	0.316 ± 0.02	0.320 ± 0.03	0.320 ± 0.03	0.303 ± 0.04	0.377 ± 0.04	0.277 ± 0.01	0.265 ± 0.02	*p* < 0.01	*p* < 0.01	NS	*p* < 0.01 (Father × Sex) *p* < 0.01 (Father × Mother × Sex)
Tau	2.36 ± 0.05	2.21 ± 0.05	2.35 ± 0.06	2.33 ± 0.03	2.36 ± 0.08	2.40 ± 0.06	2.60 ± 0.02	2.48 ± 0.05	*p* < 0.01	*p* < 0.01	NS	*p* < 0.05 (Father × Mother × Sex)
L-Ala	0.602 ± 0.02	0.554 ± 0.02	0.499 ± 0.02	0.491 ± 0.01	0.513 ± 0.02	0.518 ± 0.02	0.480 ± 0.01	0.460 ± 0.03	*p* < 0.01	*p* < 0.01	NS	NS
GABA	3.08 ± 0.10	2.82 ± 0.06	2.58 ± 0.06	2.69 ± 0.06	2.64 ± 0.11	2.94 ±0.14	2.63 ± 0.05	2.63 ± 0.10	NS	*p* < 0.01	NS	*p* < 0.05 (Father × Mother × Sex)
L-Tyr	0.092 ± 0.003	0.081 ± 0.005	0.079 ± 0.004	0.054 ± 0.003	0.090 ± 0.006	0.087 ± 0.01	0.088 ± 0.005	0.066 ± 0.003	NS	*p* < 0.01	*p* < 0.01	*p* < 0.05 (Mother × Sex)
L-Val	0.164 ± 0.005	0.152 ± 0.007	0.132 ± 0.006	0.133 ± 0.004	0.140 ± 0.004	0.151 ± 0.008	0.135 ± 0.003	0.135 ± 0.007	NS	*p* < 0.01	NS	NS
L-Met	0.605 ± 0.03	0.625 ± 0.03	0.486 ± 0.01	0.577 ± 0.02	0.504 ± 0.02	0.619 ± 0.05	0.529 ± 0.01	0.586 ± 0.03	NS	*p* < 0.05	*p* < 0.01	*p* < 0.05 (Father × Mother)
L-Phe	0.128 ± 0.01	0.100 ± 0.03	0.103 ± 0.01	0.106 ± 0.02	0.091 ± 0.007	0.107 ± 0.01	0.087 ± 0.004	0.080 ± 0.005	*p* < 0.05	NS	NS	NS
L-Ile	0.093 ± 0.02	0.070 ± 0.005	0.055 ± 0.004	0.070 ± 0.002	0.063 ± 0.005	0.066 ± 0.09	0.051 ± 0.004	0.048 ± 0.005	*p* < 0.05	*p* < 0.01	NS	NS
L-Leu	0.216 ± 0.02	0.144 ± 0.006	0.161 ± 0.01	0.14 ± 0.005	0.150 ± 0.02	0.163 ± 0.01	0.127 ± 0.005	0.132 ± 0.009	*p* < 0.01	*p* < 0.01	*p* < 0.05	*p* < 0.01 (Father × Sex)

The values for free amino acids are expressed as mean ± SEM in nmol/mg wet tissue. Here, NS means not significant; WIS♂ × WKY♀ refers to offspring of male Wistar (WIS) and female Wistar Kyoto (WKY) rats (*n* = 5–6).

**Table 2:** Effect of paternal or maternal strain and sex of offspring on free amino acid concentration in the hippocampus.

Strain	WIS♂ × WIS♀		WIS♂ × WKY♀		WKY♂ × WIS♀		WKY♂ × WKY♀		*p* Value			
Sex	Male	Female	Male	Female	Male	Female	Male	Female	Father	Mother	Sex	Interaction
L-Asp	1.83 ± 0.05	1.99 ± 0.07	1.69 ± 0.11	1.97 ± 0.05	1.58 ± 0.14	1.95 ± 0.03	1.72 ± 0.05	1.75 ± 0.09	*p* < 0.05	NS	*p* < 0.01	NS
D-Ser	0.299 ± 0.005	0.331 ± 0.005	0.266 ± 0.02	0.306 ± 0.006	0.262 ± 0.02	0.312 ± 0.003	0.265 ± 0.004	0.270 ± 0.008	*p* < 0.01	*p* < 0.01	*p* < 0.01	NS
L-Ser	0.930 ± 0.02	1.05 ± 0.01	0.832 ± 0.04	0.951 ± 0.006	0.813 ± 0.07	0.953 ± 0.009	0.824 ± 0.02	0.824 ± 0.03	*p* < 0.01	*p* < 0.01	*p* < 0.01	NS
L-Gln	5.64 ± 0.16	5.60 ± 0.07	5.01 ± 0.29	5.90 ± 0.09	4.95 ± 0.39	5.61 ± 0.09	5.54 ± 0.09	5.49 ± 0.19	NS	NS	*p* < 0.05	*p* < 0.01 (Father × Mother × Sex)
L-His	0.641 ± 0.01	0.624 ± 0.02	0.579 ± 0.02	0.605 ± 0.01	0.583 ± 0.05	0.621 ± 0.02	0.702 ± 0.006	0.595 ± 0.008	NS	NS	NS	*p* < 0.01 (Father × Mother) *p* < 0.01 (Father × Mother × Sex)
L-Arg	0.187 ± 0.01	0.219 ± 0.006	0.166 ± 0.01	0.233 ± 0.02	0.151 ± 0.02	0.232 ± 0.008	0.172 ± 0.007	0.211 ± 0.02	NS	NS	*p* < 0.01	*p* < 0.05 (Father × Mother × Sex)
Tau	6.82 ± 0.11	6.47 ± 0.09	6.12 ± 0.31	6.41 ± 0.07	5.81 ± 0.52	6.45 ± 0.05	6.48 ± 0.11	6.12 ± 0.16	NS	NS	NS	*p* < 0.05 (Father × Mother × Sex)
L-Ala	1.04 ± 0.05	1.09 ± 0.03	0.890 ± 0.05	1.00 ± 0.02	0.831 ± 0.07	0.963 ± 0.02	0.854 ± 0.03	0.831 ± 0.03	*p* < 0.01	*p* < 0.01	*p* < 0.05	NS
GABA	2.18 ± 0.08	2.68 ± 0.05	1.96 ± 0.11	2.45 ± 0.04	1.89 ± 0.15	2.44 ± 0.04	1.99 ± 0.07	2.34 ± 0.10	*p* < 0.05	NS	*p* < 0.01	NS
L-Tyr	0.092 ± 0.006	0.093 ± 0.007	0.085 ± 0.005	0.076 ± 0.005	0.090 ± 0.007	0.095 ± 0.008	0.102 ± 0.008	0.080 ± 0.006	NS	NS	NS	NS
L-Val	0.162 ± 0.005	0.152 ± 0.004	0.135 ± 0.007	0.147 ± 0.003	0.100 ± 0.02	0.144 ± 0.001	0.135 ± 0.006	0.136 ± 0.007	*p* < 0.01	NS	NS	*p* < 0.05 (Father × Mother × Sex)
L-Met	0.474 ± 0.03	0.552 ± 0.01	0.435 ± 0.03	0.505 ± 0.01	0.397 ± 0.03	0.485 ± 0.01	0.420 ± 0.02	0.431 ± 0.02	*p* < 0.01	NS	*p* < 0.01	NS
L-Phe	0.094 ± 0.01	0.094 ± 0.002	0.079 ± 0.006	0.096 ± 0.001	0.076 ± 0.006	0.104 ± 0.01	0.090 ± 0.002	0.101 ± 0.004	NS	NS	*p* < 0.01	NS
L-Ile	0.057 ± 0.004	0.074 ± 0.01	0.045 ± 0.004	0.058 ± 0.004	0.049 ± 0.005	0.054 ± 0.004	0.046 ± 0.002	0.057 ± 0.004	NS	NS	*p* < 0.05	NS
L-Leu	0.144 ± 0.007	0.156 ± 0.007	0.128 ± 0.01	0.160 ± 0.01	0.126 ± 0.01	0.154 ± 0.007	0.131 ± 0.008	0.150 ± 0.01	NS	NS	*p* < 0.01	NS

The values for free amino acids are expressed as mean ± SEM in nmol/mg wet tissue. Here, NS means not significant; WIS♂ × WKY♀ refers to offspring of male Wistar (WIS) and female Wistar Kyoto (WKY) rats (*n* = 5–6).

**Table 3:** Effect of paternal or maternal strain and sex of offspring on free amino acid concentration in the striatum.

Strain	WIS♂ × WIS♀		WIS♂ × WKY♀		WKY♂ × WIS♀		WKY♂ × WKY♀		*p* Value			
Sex	Male	Female	Male	Female	Male	Female	Male	Female	Father	Mother	Sex	Interaction
L-Asp	2.83 ± 0.11	2.85 ± 0.20	3.06 ± 0.17	2.78 ± 0.31	2.61 ± 0.13	2.39 ± 0.02	2.49 ± 0.07	2.23 ± 0.02	*p* < 0.01	NS	NS	NS
D-Ser	0.221 ± 0.007	0.314 ± 0.03	0.191 ± 0.004	0.283 ± 0.03	0.206 ± 0.01	0.209 ± 0.008	0.176 ± 0.005	0.183 ± 0.008	*p* < 0.01	*p* < 0.01	*p* < 0.01	*p* < 0.01 (Father × Sex)
L-Ser	0.841 ± 0.02	1.07 ± 0.12	0.799 ± 0.03	0.952 ± 0.10	0.756 ± 0.01	0.695 ± 0.01	0.722 ± 0.03	0.629 ± 0.01	*p* < 0.01	NS	NS	*p* < 0.01 (Father × Sex)
L-Gln	6.41 ± 0.17	8.26 ± 0.69	6.09 ± 0.14	8.28 ± 0.84	6.07 ± 0.18	6.23 ± 0.12	5.92 ± 0.07	6.12 ± 0.11	*p* < 0.01	NS	*p* < 0.01	*p* < 0.01 (Father × Sex)
L-His	0.627 ± 0.05	1.22 ± 0.11	0.544 ± 0.03	1.14 ± 0.11	0.637 ± 0.06	0.896 ± 0.02	0.669 ± 0.04	0.949 ± 0.03	*p* < 0.05	NS	*p* < 0.01	*p* < 0.01 (Father × Sex)
L-Arg	0.412 ± 0.04	0.315 ± 0.04	0.417 ± 0.02	0.345 ± 0.04	0.319 ± 0.02	0.245 ± 0.01	0.349 ± 0.02	0.161 ± 0.006	*p* < 0.01	NS	*p* < 0.01	NS
Tau	5.16 ± 0.21	6.43 ± 0.56	4.61 ± 0.10	6.20 ± 0.61	4.93 ± 0.26	4.59 ± 0.16	4.71 ± 0.10	4.46 ± 0.17	*p* < 0.01	NS	*p* < 0.05	*p* < 0.01 (Father × Sex)
GABA	7.11 ± 0.25	8.16 ± 0.80	7.04 ± 0.24	7.81 ± 1.2	6.11 ± 0.25	5.96 ± 0.14	5.86 ± 0.27	5.52 ± 0.12	*p* < 0.01	NS	NS	NS
L-Tyr	0.127 ± 0.005	0.138 ± 0.02	0.125 ± 0.009	0.117 ± 0.02	0.124 ± 0.003	0.105 ± 0.01	0.123 ± 0.006	0.082 ± 0.01	*p* < 0.05	NS	NS	*p* < 0.05 (Father × Sex)
L-Val	0.203 ± 0.01	0.221 ± 0.02	0.189 ± 0.009	0.208 ± 0.02	0.191 ± 0.009	0.172 ± 0.004	0.178 ± 0.005	0.162 ± 0.008	*p* < 0.01	NS	NS	*p* < 0.05 (Father × Sex)
L-Met	1.62 ± 0.07	1.75 ± 0.16	1.51 ± 0.04	1.57 ± 0.19	1.36 ± 0.06	1.36 ± 0.05	1.39 ± 0.06	1.28 ± 0.05	*p* < 0.01	NS	NS	NS

The values for free amino acids are expressed as mean ± SEM in nmol/mg wet tissue. Here, NS means not significant; WIS♂ × WKY♀ refers to offspring of male Wistar (WIS) and female Wistar Kyoto (WKY) rats (*n* = 5–6).

## Data Availability

Data are contained within the article.
